# Community health worker perspectives: examining current responsibilities and strategies for success

**DOI:** 10.1186/s13690-024-01313-5

**Published:** 2024-06-21

**Authors:** Monica Kowalczyk, Nicole Yao, LaToya Gregory, Jeannine Cheatham, Tarrah DeClemente, Kenneth Fox, Stacy Ignoffo, Anna Volerman

**Affiliations:** 1 Department of Medicine, University of Chicago Biological Sciences Division, 5841 S. Maryland Ave, Chicago, IL USA; 2 Department of Pediatrics, University of Chicago Biological Sciences Division, 5841 S. Maryland Ave, Chicago, IL USA; 3 Chicago Public Schools Office of Student Health and Wellness, 42 W. Madison St, Chicago, IL USA; 4 Sinai Urban Health Institute, 1500 S. Fairfield Ave, Chicago, IL #1782 USA

**Keywords:** Community health workers, Healthcare, Workforce sustainability, Logic model, Health equity

## Abstract

**Background:**

Community health worker (CHW) interventions have demonstrated positive impacts globally, with the COVID-19 pandemic further highlighting the potential of CHWs at the frontline to support prevention, outreach, and healthcare delivery. As the workforce expands, understanding the work and capabilities of CHWs is key to design successful interventions. This study examines the perspectives of experienced CHWs in Chicago about their current work and strategies for success.

**Methods:**

As part of a community-academic partnership in Chicago, semi-structured interviews were completed with individuals who held positions aligned with CHW. Interviews were conducted between January and April 2022. Questions focused on participants’ work and factors contributing to their effectiveness to gain insights into workforce strategies for success to be applied in healthcare and community settings. De-identified transcripts were analyzed using inductive reasoning with codes organized into themes and subthemes under two domains identified a priori. The themes informed a logic model focused on the early stages to support the success of CHWs in their role.

**Results:**

Fourteen individuals participated in the study. The two predetermined domains in this study were: current work of CHWs and strategies for CHWs to be successful.

Five themes were identified about CHWs’ current work: providing services, building alliances with clients, establishing and maintaining collaborations, collecting data, and experiencing challenges in role. From their perspectives, all these responsibilities enhance client care and support workforce sustainability efforts.

Five themes emerged about strategies for the success of CHWs: background of CHWs, champions to support work of CHWs, materials to perform work of CHWs, preparation for CHW role, and characteristics of CHWs. Participants described key traits CHWs should possess to be hired, individuals who can champion and advocate for their work, and specific materials needed to fulfill responsibilities. They reported that training and familiarity with the community were integral to developing and refining the qualities and skills necessary to be effective in their role.

**Conclusion:**

CHWs play an increasingly important role in enhancing healthcare delivery and improving health outcomes. This study offers a framework for policymakers, communities, and organizations to utilize for preparing CHWs to succeed in their roles.

**Table:** 

Text box 1. Contributions to literature
• This study adds to existing literature that focuses on community health worker (CHW) practices in enhancing client care.
• This study brings attention to CHW efforts in workforce sustainability, which is overlooked in existing literature.
• Our study presents a logic model, informed by experienced CHWs, on early strategies for success in CHW roles that highlights relevant trainings, materials, and support systems.
• Our research was conducted in Chicago, bringing unique insights with the city’s distinct population, healthcare systems, and communities.

## Introduction

The community health worker (CHW) workforce in the United States has grown substantially since it was established in the 1960s, with the goal of effectively linking underserved communities to vital health services [1]. CHWs—also known as promotores/as de salud, peer health educators, and lay health advocates—are frontline public health workers who help community members overcome barriers to better health and support health systems to enhance care delivery. Typically, CHWs are members of and/or have a thorough understanding of the community, improving the quality and cultural competence of services provided to individuals [2]. They provide services to both adults and children most often in community and medical settings,[3] including health education, linkages to health and social resources, system navigation, and motivation to achieve health goals [4, 5]. The potential of CHWs as key members of health care teams has been recently underscored by the COVID-19 pandemic, which uncovered and deepened health inequities. CHWs have been recognized as powerful forces for addressing the pandemic by supporting testing, contact tracing, and vaccination [6, 7].

The successes of CHW interventions worldwide are well-documented. Studies demonstrate that CHWs support individuals in managing chronic health conditions, [8–11] provide social support through screenings and referrals, [12, 13] and promote healthy behaviors [14, 15]. Such interventions are often completed via home visits and/or phone calls, through which CHWs can assess social risk factors, provide education, and support resource navigation [8, 11, 13, 14]. Additionally, CHW interventions have proven to be highly cost-effective, especially for high-risk populations [16–18].

As the CHW workforce is expanding [19] and evolving, [3] it is important to understand the current roles and capabilities of CHWs and consider factors that contribute to their success, which is critical for implementation of programs and sustainability of the workforce. While broader-scale efforts exist to understand newly acquired competencies and responsibilities of CHWs, [20] the local context may introduce unique qualities, skills, and tasks. Thus, this study aims to describe current responsibilities of CHWs and identify early strategies for CHWs to succeed in their roles from the perspectives of CHWs in Chicago in context of their unique population, healthcare systems, and communities.

## Methods

### Study design

This qualitative study was conducted as part of an academic-community partnership between an academic institution (University of Chicago), public school district (Chicago Public Schools), and community-engaged research (Sinai Urban Health Institute) in Chicago [21]. The study focused on Chicago, an urban city with several regions, including the Northwest Side with a predominantly White population, South Side with a majority Black population, and West Side with a largely Hispanic/Latino population [22–24]. This study was deemed exempt by the Institutional Review Board.

### Population

The study included individuals from the Chicago area who held positions that aligned with a CHW role, including working in and/or with a community to promote better health. Participants were recruited using email advertisements distributed by the community-engaged research institute and their partners, including various local and state listservs for community health worker organizations and resources. Interested participants then contacted the study team directly to participate. This study utilized snowball sampling with participants asked at the end of the interview to identify additional CHWs who may provide relevant insights.

### Data collection

Semi-structured interviews (*n*=14) were conducted via Zoom between January and April 2022. All interviews were conducted by a research project coordinator with a master’s degree in public health training and four years of experience in qualitative methods. Verbal consent was obtained from each participant prior to the start of the interview.

An interview guide was utilized for the discussion. Participants were asked about current work responsibilities, essential relationships, challenges faced, and methods for effectiveness in their role. This manuscript reports results from a group of questions focused on the CHW role overall; a subsequent group of questions asked about the integration of CHWs in schools and these results are reported elsewhere [25]. Each interview lasted 60-120 minutes. Participants received a $50 e-gift card. Interviews were continued until thematic saturation was reached.

### Data analysis

Interviews were recorded, transcribed, and de-identified prior to analysis. Thematic analysis was conducted based on grounded theory principles with an inductive reasoning approach applied [26, 27]. Four researchers (AV, LG, MK, NY) independently read and coded the first five interviews based on two pre-determined domains: current work of CHWs and strategies for success in CHW role. Researchers met after coding each interview to compare codes, resolve discrepancies, and develop a coding framework with themes and subthemes. Once the framework was finalized, it was applied by three researchers (LG, MK, NY) to the remaining interviews. Any new codes, themes, subthemes, and discrepancies were discussed and resolved. All transcripts were re-coded by two researchers (LG, MK) using the final thematic framework. Discrepancies were discussed until a consensus was reached. Dedoose Version 9.0.46 was utilized for analysis. To ensure validity of the results, the framework was shared with a diverse group of experienced CHWs as well as CHW program leaders, designers, and evaluators from the community-based research institute for review and feedback, which was incorporated into the final framework.

### Development of logic model

The themes from the domain about strategies for CHW success were applied to develop a logic model, a visual representation of the resources and actions needed to achieve long-lasting outcomes. This model depicts the considerations for hiring and onboarding as well as types of supports and activities needed for a CHW to be successful in their role.

## Results

Fourteen individuals participated in the study (Table 1). Their years of experience in the field ranged from 0.5 to 22 years. Some participants’ current role titles included “community health workers” and “COVID-19 response workers,” with the latter group responsible for providing resources and COVID-19 education during the pandemic. Certain participants had advanced to the roles of “CHW supervisor” and “CHW coordinator”, overseeing CHWs and programs. One participant transitioned to the role of “communicable disease investigator,” surveying infectious diseases within local communities. The majority were affiliated with health systems (*n*=11, 78.5%) and served Chicago’s West side (*n*=8, 57.1%) and South side (*n*=8, 57.1%), with some participants working in more than one region of Chicago.

**Table 1:** Participant characteristics of experienced community health workers in Chicago, Illinois, US 2022 (*n*=14)

**Characteristics**	**N (%)**
Years of experience as community health worker	
Mean (SD)	8.0 (6.1)
Median (IQR)	7.5 (9.0)
Range	0.5 – 22
Organizational affiliation, n (%)	
Health systems	11 (78.5)
Religious institution	1 (7.1)
Health department	1 (7.1)
Other community-based organization	1 (7.1)
Population/neighborhood served in Chicago, n (%)^a^	
Northwest Side	2 (14.3)
West Side	8 (57.1)
South Side	8 (57.1)

^a^Total number exceeds the number of participants as some participants reported serving more than one Chicago region

### Domain 1: Current work of CHWs

Five themes emerged from participants about the current responsibilities of CHWs: providing services to clients, building alliances with clients to improve health outcomes, establishing and maintaining collaborations, collecting data to support work, and navigating challenges in their work (Table 2).

**Table 2﻿:** Themes, subthemes, and illustrative quotes related to current work of community health workers (CHWs), as informed by interviews with experienced community health workers in Chicago, Illinois (2022)

**Theme**	**Subtheme**	**Illustrative quotes**
Providing services	Services for clients	"With lead [project], it's just a bunch of phone calls [with clients] and asking a lot of questions. And then hoping that they're aware of what lead is…Education is everything. So that's practically what I do.” (Interview 5)
		“I [CHW] follow up with them [clients]. After they are discharged [from Emergency Department], I make sure they’re okay with medications, food. Sometimes they say, ‘I might need another appointment.’ I try to make another appointment. Sometimes the [contacts for] resources are not answering, so I’ve got to call them. It’s the whole process - make sure you have done your job right. When you provide resources, you’ve got to make sure they [clients] are using those resources.” (Interview 8)
		“We address a lot of things. We address transportation to doctors’ appointments…We talk to insurance companies. It’s all on what the patient needs. At the beginning of the year, people’s deductibles have started over. They’ve been running into issues at the pharmacy trying to get prescriptions filled, and so there’s been a lot of workarounds with that.” (Interview 11)
		“Tomorrow I’m going out to deliver what we call green clinic kits…they [clients] could get these free products… we’re not doing a lot of home visits because of COVID… So I’m just going to go to the homes and get the HIPAA documents signed and drop the kit off at the home.” (Interview 12)
	Services for program development and implementation	“Being more of a team lead [and] making sure that the CHWs on our teams have the support that they need to be successful in their roles [are my current work responsibilities]. I have a project where I’m trying to get courses or presentations to be available to CHWs as we move into different roles or things they want to get a refresher on. Being more of a mentor to the newer CHWs.” (Interview 1)
		“When it's a new program, it's rare if things come in translated. Or when you're getting trained as well, a lot of things you don't get as trained in Spanish because they're training you in one language and that's it… I went ahead and looked up and translated these documents.” (Interview 5)
		“I help with program design, refinement, and development. That will apply to most of the projects I work with. For diabetes prevention [program], I set up and establish my cohorts - recruiting them [clients], establishing a schedule for us to meet, collecting materials and resources to distribute to them, doing the presentations, doing data collection, and managing that.” (Interview 6)
	Services for community	“We do a lot of outreach in the community. During October, we do workshops, presentations around breast awareness. I would say mostly just empowering people to get their screeners done.” (Interview 7)
		“We went to several schools. While we were there and if they wanted some information on the vaccination for their kids, for their parents, you let them know what you’re doing.” (Interview 10)
		“We [CHWs] participate in health fairs no matter where it’s at. I’ve been to Englewood to health fairs, different activities that they have for the community and we try to make ourselves known in those settings, so we interact with health fairs, different activities.” (Interview 12)
	Support for clinical team	“I collaborate with physicians to document and for clarity what they [physicians] want and expect out of the patient. Do I need to verify information like medications or the last doctor appointment [and] the next doctor’s appointment? How would you like me to educate this person? Because we [CHWs] are the eyes and ears of the physician.” (Interview 2)
		“Being a CHW, we’re kind of like the liaison between the patient and the physician. We find that a lot of times our clients [are] not comfortable speaking and being open with their physicians as they are to us. It’s our job to listen to them and then relay the message back to the doctor’s office.” (Interview 12)
Building alliance with clients to improve health outcomes	Steps for establishing relationships with clients	“I introduced myself or even let them [clients] ask me question. Is there something you want to know about me? Or else [I] share something, like without them having to ask. Or I'll be like, ‘Oh my son really likes that.’ And they're like, ‘Oh, you have a kid?’… you don't always want to just ask some questions right away without getting to know them a little bit better.” (Interview 5)
		“At at the end of the day, if I was asking questions and if the person wanted to stop me and talk about something pressing… I had to take a step back…So those are the things that we just had to be better at and continue to build relationships with people.” (Interview 7)
		“A lot of them [clients] don’t understand why you’re there. ‘Why are you here? Are you going to call the DCFS?’… once they understand that you’re just there for health reasons then they open up to you a little bit more.” (Interview 12)
		“There was one instance where I knocked on her [client] door. She was in her late eighties…They were about to evict her from her place. She didn’t know how to fill out the paperwork. I was able to give her some information and to just let her know places that she could call. That’s the only way, I mean how else do you do that outside of talking to people? You initially have to talk to them and find out what their issues and needs are.” (Interview 14)
	Interactions with clients	“You make these engaging connections…Those connections are made virtually, but typically [would] be in that person’s safe space. Having that time to talk with them… to see what’s the root cause of what’s going on and then try to address that so it can change some of the other outcomes.” (Interview 3)
		“Our first communication is over the phone and then we do an intake. There's been instances after we schedule the appointment, patients are pretty vocal as to, ‘Can you text me? Don't mail me a letter because it will get lost. Maybe text me two days in advance.’ We do texting two days in advance. Some people want reminders the day before and the day of. Some people want a letter, so we mail them a letter. Some people would rather just have phone calls. We do that.” (Interview 7)
Establishing and maintaining collaborations	Collaborations within health systems to support clients/patients	"We're part of a larger health system, we get to do internal collaborations across departments and divisions as well, such as doing integrating and deploying CHWs in the emergency department to help support patients with social determinants of health and getting connected to resources and support.” (Interview 6)
		“I'm thinking of a pilot that we have for oncology where we had a social worker and a CHW and then the nurse navigator… three people that had different distinct roles… case worker was busy with other patient, so then she would send an e-mail to the educator [CHW], ‘Can you please call this patient?’ She needs this and that and you connect her to transportation. So delegating things that could be done by not necessarily a nurse but by a CHW.” (Interview 7)
		“At the hospital, I go to the in-charge nurse. She knows how is the ED running, so she will be able to help me. She will be able to refer people [to the CHW], so that’s how we collaborate. This is new for this hospital - all this project is new. They don’t know exactly what resources or what are my connections or what can I do for a patient.” (Interview 8)
		“We have a very close relationship with social work. When we first started integrating CHWs into the health system, they were the first people that we contacted and they allowed us to walk the floors with them. We got to see how they screen people, how they identify needs with people, and then they kind of just threw us in. We got to talk to them, so we collaborated with them a lot.” (Interview 11)
		“When I first started as a CHW, a lot of people weren’t aware of us. Right now, we are popular. The clinicians are usually open and willing to help you.” (Interview 12)
		“We have a relationship with them [clients’ providers] because we’re in a constant meeting with them updating information about cases.” (Interview 13)
		“You need to communicate [with clinicians]... Sometimes it can happen that they make the same appointment, or they forget to make the appointment so it’s [important] to keep them updated about what resources you are providing.” (Interview 8)
	Collaborations with community health worker team	“Usually we have a monthly debrief as well with just CHWs, which helps. They usually are bimonthly when a program starts. Once the program is rolled out for a bit and we see it's flowing, we decrease it to once a month. We give that space to CHWs to debrief and tell us how they're feeling, what's working, what's not working, and see what we can do to change some of those.” (Interview 5)
		“We [COVID response workers] work together. We talk about the [vaccine] pods every Thursday. We talk about the numbers of people that came and got vaccinated that day. We talk about what can we say different. So it’s always collaborating with each other because of the type of job we have.” (Interview 10)
		“We work on different projects if it’s easier. ‘I have an asthma patient. Could you talk to them? Could you do education?’ I’ll just call them and then they’ll come do the education.” (Interview 11)
		“If it is something I don’t feel that I’m dealing with correctly, our CHWs are so open to assist you. We help each other.” (Interview 12)
	Collaborations with community organizations	“We’ve collaborated with community organizations like to help their CHWs get trained on the trainings that we offer… like [external organization]…we are able to refer some patients to them.” (Interview 1)
		“Some [clients] fall out of your area of expertise or even a language issue. You want to get them to the right connectors. We have beautiful relationships with our external partners, different organizations that do some of the same community work with CHWs. If it falls outside of our area of expertise or our area of help to reach, we definitely refer them to our partners in this. We’re able to create that warm hand-off.” (Interview 3)
		“I work with community councils to have our hand on the network of community organizations that are providing services. Anywhere people are we will try to get in there if they want community resource service support.” (Interview 6)
	Collaborations with community members	“I also do intentional networking with the neighborhood to help facilitate and foster community relationships. I sit on an array of committees and boards.” (Interview 6)
		“We do have a community advisory board. Whenever we want to do changes to our programs, we try to connect with them to see what they say and one of those is an example of our name. We're trying to be more inclusive and less gender. More gender neutral. We went to our advisory board, which is encompassed by community members to figure out what they recommended. So those are the type of collaborations we have with the community.” (Interview 7)
	Other collaborations	“I do collaborate with Chicago Asthma Consortium, also with the Illinois CHW network. Matter of fact, I’ve got a meeting coming up with them later on. Those are some of the external partners that I collaborate with sometimes daily, sometimes weekly or monthly. My go-to people by email like in webinars is allergy and asthma. That’s my go-to for resources and information.” (Interview 2)
		“Joining the national CHW organization - that’s a must because you’re getting information.” (Interview 13)
Collecting data to support work	Health and social data of patients/clients	“With our program, we do use REDCap. One of my newer responsibilities is once a social worker refers patients to our program, I have now taken the responsibility of entering that data into REDCap. Their demographics is entered and what social worker referred them to the program. That is some information that I enter. Once I’m working with a patient, I enter the notes - what happened during our conversation, what support do they need.” (Interview 1)
		“It's part of -- for the program for prediabetes. It's a requirement for CDC, for us to report that. So we always need to track weight and their physical activity.” (Interview 5)
		“That information [data collected] allows us to see what they’ve [clients] needed assistance within the past. It allows us to see if those were addressed. It shows us readmission data. If this person is frequent in the ED, I can see each time that they were referred to us. I would see whether they want [CHW] assistance or not.” (Interview 11)
	Data for tracking work progress	“All the information that we capture is entered in there [REDCap] and our data manager’s able to create reports. Usually it’s a monthly report, so we’re able to see how effective we are if we are doing what we’re supposed to be doing on our end.” (Interview 1)
		“We enter the resources they need and if we provide it. Sometimes, they [clients] ask me for a passport replacement. On my notes I say, ‘I referred a patient for passport replacement here,’ and I enter notes because sometimes you have bad experiences. You enter information there.” (Interview 8)
	Data for evaluation of program and services	“Overall, it’s [the data is] used to let us know what resources are still active and actually helpful. During the pandemic, a lot of the resources we had before either vanished, no longer serving the community, or ran out of funding and things. It helps us to keep our resources active.” (Interview 11)
		“We are the research arm of the hospital, so a lot of our information is taken to determine what’s going on in different areas [and] the outcome of different areas.” (Interview 12)
	Data for sustaining and expanding efforts	“It [data collected by CHW] gets reported to the system as a whole to evaluate programs and the initiatives to move forward or broaden, which is why we’re at the point we’re at with CHWs within our own organization. The healthcare system were able to see those results from those previous interventions and see the effectiveness of the [CHW] role. Now, it’s been the commission of the system to infuse CHWs through all departments.” (Interview 3)
		It [the data] was also utilized to get more grants to bring more sustainability to some of these programs. Rather than waiting on grant funding but building a mechanism that could employ CHWs and keep them as a part of the team and not just drop off once the funding is ended.” (Interview 3)
		“I was in a diabetes program. Before I started, it was called [program 1]. Once they collected that information, they saw some fine-tuning they needed to do and so they did it. Then, they created a second one, which was called [program 2]. That’s when they hired new CHWs. They brought in more because they realized they needed more CHWs to roll out this program.” (Interview 5)
	Data for dissemination	“We use [data] to improve our programs. Use it to help tell the story of these community concerns we were addressing or identified through this programming. Data is our support and validation. It’s very important, and very true to our nature and spirit as an organization.” (Interview 6)
		“I'm thinking about the asthma program. In the past, we got published. We were able to get published and figure out the cost savings that the hospital was having by utilizing the CHW.” (Interview 7)
		“It’s [the data is] trying to give knowledge to the medical field, whether the district or area of what we really need to focus on. It’s trying to teach us what we really need to focus on and how to deal with people with certain issues and how to provide the things that they need.” (Interview 12)
Navigating challenges faced by community health workers	Challenges within relationship between community health worker and client	“In the beginning, it was very hard for me to set boundaries. I would cry with my participants. I would hold their hands. Then they're calling me in the middle of the night asking me all other kind of stuff. I'm just like, ‘oh no, what did I do?’ And so, you learn from those lessons.” (Interview 6)
		“In the past, it was a struggle to get that trust from the community. The health care system has been very harsh towards black and brown individuals. A lot of studies that affected many lives and to this day have created a lot of mistrust.” (Interview 7)
		“We’re still going through a hard time. We’re still experiencing people who not only won’t get vaccinated, but don’t want to learn, don’t want to get the accurate knowledge. It’s frustrating. It’s a challenge to stay positive when you know this [vaccines] has got to be helpful, it’s going to prevent someone from being sick, it’s going to prevent someone from dying and then to have someone still tell you absolutely not. That’s a challenge.” (Interview 14)
	Resistance to integration in clinical areas	“The world doesn’t see a CHW like they would a CNA [certified nursing assistant] or another kind of assistant. It’s like an ad hoc piece. We know in doing the work that we’ve done, it’s been verified and documented that having a CHW attached to a person within their health challenges makes a difference, and we know for every dollar spent in some programs, you can save as much as seven dollars per person. That’s always a win-win.” (Interview 3)
		“This project put community health workers into ED. It was trying to prove how we were needed in the emergency department. It sounds easy, but at first it was very, very, very difficult to get them acclimated. Because nurses [and] doctors were very difficult to accept us. We weren't there to take their role either.” (Interview 5)
		“The other part of the [clinical] team don't let you fit in. They maybe don't understand. It is community health - it's health, so I'm part of this… then that make you feel like oh, okay… [they] want to say that I'm the noisy person of the clinic? Yes, I am, but it is the way I can help my community: being noisy.” (Interview 9)
	Challenges related to materials needed to perform role	“Part of our program is giving them [clients] incentives. We can only order a small amount per time. When we're starting a new cohort, sometimes we have to start without those incentives. They're actual tools that they need to keep using for the program. That's mainly one thing that comes up when we need something, and we have to order it and we have to wait for that delivery time.” (Interview 5)
		“A stipend or support to the shift of working from home. I think that we take for granted employees who work in remote locations to do work. That offsets our at-home utility bills. Now that we're utilizing our own utilities to support our work efforts, some type of bonus or stipend to support utility.” (Interview 6)
	Gaps in resources	“Our clinics refer less because with the pandemic - there's less visits to the doctor. It's been a struggle.” (Interview 7)
		“With COVID-19, a lot of people lost their employment. I think the financial piece from my end and the kind of work that we do is a struggle because people have to really think about it. If there’s no resources, then it’s hard for people to commit to having a procedure in a hospital setting where they know they’re going to get a bill that they might not be able to pay accordingly.” (Interview 7)
		“There are resources for people up until 18, and then there are like resources for people starting at like 26. What happens to the people in between or people who need resources from like 26 to 54 when you have to be 5? It’s weird age gaps. People who have been incarcerated [and] have things on their record from 20 or 30 years ago are still preventing them from getting resources now. That’s really difficult.” (Interview 11)
	Frequent changes/adaptations in work	“We started some projects that we wish would have turned out differently. Because it consisted of home visits. Now it's more difficult to get them [clients] virtually and actually show you [their] home. As opposed to first building that relationship with them and allowing them to let you go in their home. You would think it's easier virtually because you're not going in, but they don't want to do it.” (Interview 5)
		“We live in Chicago. When it’s so cold, we don’t have that much patients. After a very cold day, we have big amount of people with fractures or stuff like that. When the spike of COVID was high, we were struggling with stress because people were overwhelmed and they had to wait for a long time. Every day, it’s different.” (Interview 8)
	Other challenges related to scope of work	“I think one of the challenges is not bringing it home with you, but we are home now… Don’t carry it because that can become overwhelming… Some patients really do need support and they want to stay with you on the phone for hours and we can’t do that. Then there are some that we are in the process of helping and they don’t make it. They pass away, so I think those are very difficult situations and, you know, learning how to destress and not feeling guilt maybe or knowing that we did our best that can be difficult when you have situations or cases like that.” (Interview 1)
		“That’s one of my biggest problems, street safety. Most of the neighborhoods that we work in are high crime areas… It wasn’t as bad as it is now and just eight years ago. Eight years ago is not such a long time, but it was then when I first started we could make our appointments for early in the morning and it’s usually safe in the morning, but now it’s just as bad early in the morning as it is in late evening.” (Interview 12)

#### Theme 1: Providing services

Participants emphasized their primary role is to provide services to clients seeking care for their needs. Examples of services included navigation of the health care system and linkage to health and social resources. Participants reported additional services for clients included educating about health topics, delivering materials (e.g., green cleaning kits, medications), and troubleshooting issues negatively affecting care – all of which are addressing “what *the patient [client] needs.”*

Participants also shared their contributions to the development and implementation of CHW programs, including supporting design of programs and materials. CHWs applied their unique expertise to developing projects. For example, one participant shared, *“Sometimes, especially when it’s a new program, it’s rare [to have] things come in translated; or when you’re getting trained, you don’t get trained in Spanish. You have to do your own translation.”* Participants described additional responsibilities including conducting recruitment, data collection, and trainings. One participant described that they ensured continuing education was available, *“I’m trying to get courses or presentations to be available to CHWs as we move into different roles or things they want to get a refresher on.”*

In specific settings, participants described CHWs providing more tailored services. They discussed conducting health outreach and education at community events, such as health fairs. Although the COVID-19 pandemic paused direct outreach, some participants still conducted community outreach including canvassing schools to provide COVID-19 information: “*if they wanted some information on the vaccination for their kids, for their parents, you let them [schools] know what you’re doing.”* In clinical settings, CHWs can uniquely connect with clients to further learn about their health status and factors influencing their health to inform clinicians as one participant explained, “*a lot of times our clients [are] not comfortable speaking and being open with their physicians… It’s our job to listen to them and then relay the message back to the doctor’s office.”*

#### Theme 2: Building alliances with clients to improve health outcomes

Participants described that, for CHWs to effectively provide services, a significant part of their work is establishing trust with clients. They emphasized the importance of CHWs utilizing their communication and interpersonal skills by being attentive to clients’ needs, connecting with clients through shared experiences, being transparent with clients, and assuring clients of their support. One participant shared how they first interact with clients, *“I introduced them myself or even let them ask me question… Things like that have also been very helpful in building that rapport with the participants.”*

Additionally, the various ways that CHWs interact with clients can help establish alliances. Participants expressed that in-person contacts through door-to-door canvassing and home visits effectively built trust with clients. While the COVID-19 pandemic resulted in limited in-person interactions with clients, one participant emphasized the importance of continuing meaningful engagement with clients in virtual environments, *“Having that time to talk with them and to ask those opening questions to see what’s the root cause of what’s going on and then try to address that so it can change some of the other outcomes.”*

#### Theme 3: Establishing and maintaining collaborations

Participants also described how their current work leverages their various relationships with partners: collaborators within health systems, CHW teams, community organizations, and community members.

Participants affiliated with a health system described working across departments and collaborating with various personnel to support patients. They indicated the integration of CHWs into health teams has become an easier process with health systems becoming more familiar with CHWs as one participant shared.*“When I first started as a CHW, a lot of people weren’t aware of us. Right now, we are popular.”* Further, some participants explained that they worked directly with clinicians in the health system to coordinate care for clients. One participant emphasized the importance of consistent communication in this partnership, *“Sometimes it can happen that they [clinic staff] make the same appointment, or they forget to make the appointment so it’s [important] to keep them updated about what resources you [CHWs] are providing.”* CHWs also relied on collaborations within the health system to acquire knowledge and resources. One participant shared about their partnership with social workers, *“[Social workers] know a lot. If they don’t know or if I don’t know, they’re finding the best way to figure it out.”*

Beyond the health system, participants shared that it was essential to collaborate within their CHW teams. Participants expressed these partnerships have helped them be effective and stay motivated in their roles, especially during a public health crisis. Some participants shared that, even with limited in-person interactions, they made time to meet with their coworkers to share successes and challenges as one participant explained, *“If it is something I don’t feel that I’m dealing with correctly, our CHWs are so open to assist you [me].”* Additionally, through these partnerships, CHWs were able to share their expertise to support each other’s clients. One participant described, *“We work on different projects… ‘I have an asthma patient. Could you talk to them? Could you do education?’ I’ll just call them and then they’ll come do the education.”*

In addition to working with health systems and CHWs teams, CHWs discussed their collaborations with community organizations, community members, and other partners (e.g., CHW associations and groups) to gather and expand resources. CHWs worked to develop partnerships with local organizations and external networks to link clients to resources (e.g., food pantries, housing assistance) as well as to provide health education to community members. One participant shared the benefits of having such local partnerships to minimize gaps in resources, *“If there’s one [service] that we don’t offer, then [we are] reaching out to organizations that offer [the service].”* Participants described that these partnerships can be bidirectional with CHW providing trainings to these organizations. Also, participants stated CHWs worked closely with community members to tailor their services to community needs. For instance, CHW programs have organized community advisory boards to obtain suggestions to enhance the programs. One participant shared how they leverage their community advisory board to enhance a CHW program, *“We do have a community advisory board. Whenever we want to do changes to our programs, we try to connect with them to see what they say and one of those is an example of our name*. *We’re trying to be more inclusive and more gender neutral…so we went to our advisory board.”* Additionally, participants have worked with CHW networks to build knowledge and expertise as one participant shared, *“Joining the national CHW organization – that’s a must because you’re getting information.”*

#### Theme 4: Collecting data to support work

Some participants described significant involvement in data collection as a part of their roles. They stated that CHWs collect health and social-related data to identify factors influencing health outcomes as well as to monitor client’s progress in working with a CHW. Additionally, participants shared the importance of data collection for CHWs to track their own work progress. This data may be related to the reach and impact of their work, including the number of client interactions each day and number of clients who accepted or rejected services, resources, or referrals. Participants also talked about the importance of tracking outcomes of resources provided to clients to screen for the quality of services provided. One participant described the information they provide, *“We enter the resources they need and if we provide it… and I enter notes because sometimes you have bad experiences.”*

Participants indicated the data collected by CHWs are utilized for evaluation, maintenance, expansion, and dissemination of programs. Data on the outcomes of CHW services can highlight the successes of CHW programs as well as areas for improvement. For example, one participant shared how the data collected led to not only refining the program but also expanding it, *“I was in a diabetes program. Before I started, it was called [program 1]. Once they collected that information, they saw some fine-tuning they needed to do and so they did it. Then, they created a second one, which was called [program 2]. That’s when they hired new CHWs. They brought in more because they realized they needed more CHWs to roll out this program.”* Additionally, this data can bring to light the needs of vulnerable communities and impacts of CHWs through dissemination: *“We use [data] to improve our programs. Use it to help tell the story of these community concerns we were addressing or identified through this programming. Data is our support and validation.”*

#### Theme 5: Navigating challenges in role

Participants highlighted various challenges encountered in their community health work. One challenge was related to the different relationships within their roles. CHWs shared difficulties in their client relationships. Some participants discussed working on overwhelming client cases and coping by learning to create healthy boundaries and rely on other CHWs. CHWs also spoke about barriers in interacting with clients, especially virtually, which can limit communication. Participants shared that some clients may be heavily reliant on CHWs, while some may distrust them: *“In the past, it was a struggle to get that trust from the community. The health care system has been very harsh towards Black and Brown individuals.”* Beyond client relationships, some participants described challenges with integrating into clinical teams. Some experienced being unwelcomed by clinicians and other staff due to limited understanding about CHW role. One participant cited the insufficient recognition given to the work of CHWs, “*The world doesn’t see a CHW like they would a CNA [certified nursing assistant] or another kind of assistant... it’s been verified and documented that having a CHW attached to a person within their health challenges makes a difference.”*

Another challenge described by participants was related to gaps in resources for clients and materials needed to complete their work. Participants highlighted the limited funding to support clients and CHW programs. One participant described the struggles of overcoming financial barriers for clients to receive health care, “*With COVID-19, a lot of people lost their employment… If there’s no resources, then it’s hard for people to commit to having a [medical] procedure.”* Additionally, there are limited funds to provide as incentives to foster success in CHW programs.

Additional challenges raised by participants included fluctuations in work. They indicated the pandemic led to drastic changes with the transition to remote work, which affected how CHWs interacted with clients. One participant shared, *“Now, it’s more difficult to get them [clients] virtually and actually show you [their] home... You would think it’s easier virtually because you’re not going in, but they don’t want to do it.”* With remote work also comes the challenge of maintaining a work-life balance that participants reported, as one shared, “*Some patients really do need support and they want to stay with you on the phone for hours and we can’t do that.”* Lastly, participants discussed how the nature of home visits and community outreach posed a challenge due to increases in crime in the communities served: *“Most of the neighborhoods that we work in are high crime areas… We could make our appointments for early in the morning and it’s usually safe in the morning, but now it’s just as bad early in the morning as it is in late evening.”*

### Domain 2: Strategies for CHWs to be successful in their roles

**Fig. 1:**
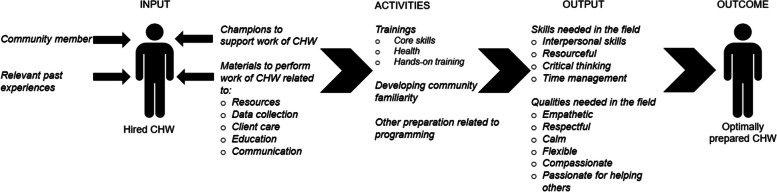
Logic model describing strategies for community health workers to succeed in role, as informed by interviews with experienced community health workers in Chicago, Illinois (2022)

Five themes emerged from participants about strategies for CHWs to be successful in their roles: background, champions to support work, materials to perform work, preparation for role, and acquired characteristics (Table 3). These strategies aligned with a logic model, including inputs, activities, and outputs with the outcome of an effective CHW (Fig. 1).

**Table 3﻿:** Themes, subthemes, and illustrative quotes about strategies for community health workers (CHWs) to succeed in role, as informed by interviews with experienced community health workers in Chicago, Illinois (2022)

**Theme**	**Subtheme**	**Illustrative quotes**
Background of community health workers	Community member	“A lot of our CHWs are really from the community and know how to navigate people in the right direction.” (Interview 7)
		“What they [the program] try to do is to get a person from the community already because the clients really trust us because we look like them.” (Interview 12)
	Relevant past experiences that prepare for role	“I wanted to be in the health field because my grandmother was in the field, and I saw how she took care of patients. It was a point where I had to take care of her. That gave me the opportunity to take care of other people despite what health issues they may have.” (Interview 4)
		“I had my first job out of college with the Chicago Coalition for the Homeless… I already had that experience as far as engaging the community and doing that door-to-door situation. I think that helped me get [first role]... Sinai has always been a hospital of social justice, dealing with the disparities. We were already doing the work 20 years ago.” (Interview 7)
		“I think having a previous knowledge like myself, my background in psychology had helped me a lot. I also have experience, you know, like talking to people, dealing with stressful situations… you need to have like a background experience like social studies or psychology or something related to health and mental health.” (Interview 8)
		“I was the mother waiting in a room to get the service, and nobody gave the service because [there was] nobody who speaks Spanish. I wait from 7:30 in the morning through 4 o’clock in the morning waiting [for a] translator. I'm not the first, but I'm not the last one. That gave me that responsibility.” (Interview 9)
		“We have a list of food pantries and we also use NowPow that will direct us to the nearest food pantry, shelters, PCPs, help with doctors’ appointments. It’s just a list of things. It might be something that one client asked me for that the next CHW has no idea of where this is. It might just be something a family member experienced and I was like, ‘Oh, okay, you could go here because my aunt went there. So it’s a range of things that we help with.’” (Interview 12)
		“I have worked in social services for so many years, I have worked with a different environment than aside from this [COVID-19] virus, but where people have suffered loss. It’s opened me up to be more empathetic, more understanding, more patient, more willing to, as I said, listen rather than speak, more open to giving rather than take, take, take.” (Interview 14)
Champions to support work of community health workers	Support from internal organization	“When I got into the space, them [the organization] readily having support available, them training me to fulfill the expectations that they have for me, that helped me.” (Interview 6)
		“I think one of the reasons that I really like to do what I do is because I feel supported and because, like [organization’s] philosophy, if we’re okay emotionally, we can better perform our job.” (Interview 8)
		“Our organization makes sure that we’re prepared and have everything that we need. If it’s something that comes up that we don’t have, we just go to the administration and they make sure that we have it.” (Interview 12)
	Support within the CHW team	“It could be overwhelming [in the beginning of role]… something came up that was so bizarre… I’m like, ‘I can’t do this. I’ve got to find something else to do.’ My [team member] was like, ‘Okay, see you tomorrow’ … I can laugh at it now. When people new come to me, I’m like, ‘Okay, let’s take a deep breath. Go home and do some self-care’ … It’s vitally important to sit under someone that has the experience.” (Interview 2)
		“I love that we [CHWs] get an opportunity to have one-on-ones and then group sessions… First of all, we’re able to decompress. We’re able to say what’s going well, what could use a little improvement, and then how we can stay motivated because this time in our lives has brought us a lot of challenges. Life is happening right alongside COVID. Other things are still going on.” (Interview 3)
		“Visiting someone’s home could be a really big shock to you when you first walk to the door… you have to go in really calm and compassionate, so the support of your coworkers and your supervisor or your trainer [is necessary].” (Interview 12)
	Support from leadership within community health worker team	“I was doing a health fair one summer…There was a shooting. I saw somebody get shot. I was just in total shock and I didn’t realize it affected me that much. When I shared that with my supervisor, she was like, ‘That’s a lot to deal with. What is it that you need to support you with handling what you saw?’…When we would do our weekly check-ins, she would always ask about how are you doing. That… allowed me to continue to do the work.” (Interview 3)
		“I’ve had supervisors that didn't advocate for me. I [felt] like I couldn't even go to them...even looking my calendar, they wouldn't respect it. Having that type of support with your supervisor does make that type of work a lot easier...it makes [me] happy to be working in that field.” (Interview 5)
		“One you start working, you realize, ‘oh, I need this and I don’t have it’… anytime I need something I just text my supervisor and… They’re very wonderful people, so they called the supervisor at the hospital… they let them know…’the CHW needs these resources or needs this.’ But yeah, you will find certain things that you don’t have on hand and you need them.” (Interview 8)
		“I feel very blessed to have the support of [supervisor]. We would have a meeting every month to say what else do you want to do? Or why do you think it will work and get it to the next stage?” (Interview 9)
	Support from external organizations	“We have a hub of five different organizations that operate in different disciplines. We all get together to collaborate as CHWs to talk about experiences… and we’re able to support one another. If I come across somebody who’s dealing with being unhoused [and] I have a partner that works at homelessness prevention within my network, I’m going to say, ‘I have a person, this is their name. I’ll tell them to call you.’ It breaks down the silos.” (Interview 3)
		“I have this one champion. I had her and she was freaking awesome… she is a doctor still. She is the one that was, ‘go ahead and talk [to] them,’ ‘go ahead and do this,’ ‘go ahead and tell them.’” (Interview 5)
		“A lot of times clinicians tend to be a bit territorial with the CHWs. We do have a lot of champions out there that believe the CHW model is effective. It should be a team effort making sure that the rules are clearly aligned and reflected on whatever process you're going to have.” (Interview 7)
Materials to perform work of community health workers	Resources to services for clients	“When I provide their resources to patients, I need to make sure they understand instructions because sometimes they don’t know what to do [or] where to call when… That’s why I have physical material, physical resources. I can get copies and highlight numbers they’ve got to call, addresses, appointments. I will make sure they understand what they have to do to get the resources they need.” (Interview 8)
		“NowPow is a really great tool because it shows you resources that are in specific zip codes. You can search for whatever it is that you’re looking for and it’ll show you what’s there, what’s still active and it lists requirements and qualifications.” (Interview 11)
	Materials for client care at their home	“We definitely have the items that you utilized when we are in person with the client or providing services with them. If it is additional medications that they need, then we are able to help facilitate with that. We get that.” (Interview 3)
		“When we were doing home visits, we had asthma kits that we would take out. They contained green cleaning products. They contained sometimes vouchers. They contained pillowcases that were like hypoallergenic… depending on like what you’re going to see a patient for, your materials could vary.” (Interview 11)
	Materials to facilitate communication	“I think the Cancer Society's pretty good about having enough material both in English and Spanish. We are seeing a high number of Middle Eastern women. We did translate our HIPAA form when we enroll them. I haven't seen anything in Arabic. Something for the Middle Eastern population would be ideal for sure.” (Interview 7)
		“The system that we use the EHARD, we get personal messages... A lot of [clients] have that social media account. Different ways to contact them as long as we have a contact because you can’t really discriminate when you’re trying to stop the spread [of infectious diseases].” (Interview 13)
	Materials for data collection	“We've been doing this [CHW] project since 2007 so you can imagine the amount of records that we have. We decided to start a REDCap database. It's easier for reporting purposes, for funders, and for reporting to our hospital boards and stuff.” (Interview 7)
		“We have iPads to enter data with if we need to do that. We also have our laptops and computers to do that work for the most part.” (Interview 3)
	Educational materials	“When we were doing diabetes education, we would take these little patches to teach them [clients] how to stick themselves. We would take some like lists of healthy foods on it. We have binders with information about who to contact if you have questions, how to set up a doctor’s appointment, and what your sugar levels should be.” (Interview 11)
		“At train.org, they had us [CHWs] doing a lot of classes. I probably received maybe eight or nine certificates. On Coursera, I probably received 11 [because] we had to do a lot of courses on those, so that gave us a lot of information.” (Interview 10)
Preparation for community health worker role	Community health worker core skills training	“I had to understand cultural humility…I didn’t know how to be a part of the multidisciplinary team [and] how to document. I needed to understand what is it. Once the expectation had been set and the knowledge has been layered on that, then it gets you ready to do the work in hand.” (Interview 3)
		“I asked for additional layer of training to get acclimated to the [CHW] role. That was ACES training which really helped me. It helped me to understand that the people who were in front of me [were] not just a culmination of them at that moment, but a process to who they are. It just gave me a different lens and allows me to lead with compassionate empathy first.” (Interview 3)
		“We have to make sure that we asked the question as it was in the paper but making it conversational or not just reading it robotically but having that eye contact, being comfortable. My demeanor while practicing… I'm not showing any emotions when I'm asking questions and I get an unusual response. People know when you're not genuine and that's something that I learned my body language and being open, not closed.” (Interview 7)
	Training on health topics	“All the CHWs were cross-trained, so we all have training in asthma. We all have training in diabetes…If we’re not in the program, we’re also trained to be backups in case a CHW takes time off… For our role in the CHW support program, we have all kinds of patients, COPD, or diabetes. So that also helps us be more knowledgeable and be able to assist the patient more.” (Interview 1)
		“We can all jump in different interventions and educate a person. If I’m seeing somebody for breast health and I know that person has asthma, I can interject some of my asthma knowledge.” (Interview 7)
		“We definitely did mental health, first aid training. We did CPR, basic lifesaving things… We did trainings on asthma, how to administer the Albuterol. We did trainings on diabetes, how to test blood sugar, what those numbers mean. There were so many trainings.” (Interview 11)
	Hands-on training	“Trainings, a lot of trainings [prepared for CHW role]. When I first started, it was mainly diabetes. I had extensive training with different diabetes programs that were out there. Also, that hands-on experience - I was shadowing other CHWs, going out into the field. That helped a lot as well.” (Interview 1)
		“We had our asthma education training with like 176 hours of training. We went through role plays. We went through shadowing and I was not released to do it by myself until I continued with this entire process. But the doctors that were attached to the program gave us the appropriate curriculums and protocols to follow. Then the leaders that were, my supervisor gave the training and took me through the test.” (Interview 3)
		“CHW is something that you learn on the job. You can have a lot of certificates [and] a lot of workshops. If you don't have that experience from doing home visits with the educators, outreach, having face-to-face contact with people… you're never going to have that connection with people. I'm so grateful that [organization] was able to allow me to see that.” (Interview 7)
	Ongoing professional development and continuing education	“The learning part is what has helped me. If I ever saw something that would lend to me being a better version of myself as a CHW, I wanted to know more… If I brought up to my supervisor, I saw something that would better help me do my role, I had their support and was able to take part in that.” (Interview 3)
		“They [CHW organization] really care about you. They provide me a lot of courses [and] a lot of training. They have all these programs for continuing education and improving your skills as a CHW.” (Interview 8)
	Developing community familiarity	“What was most helpful for me in doing the work? Being able to give back because I did grow up in the West Garfield area. Being able to identify that there were many opportunities for me that I was able to get out of in the community. To be able to help in service other people in the community is what I was grateful for.” (Interview 4)
		“I’m originally from Georgia. When I moved here...I wasn’t familiar with the areas... some neighborhoods are safer to be at on certain days and times. That was the information that I had no knowledge of. When I started [as CHW], I just wanted to make sure that I was safe physically because you can’t help anyone if you’re not around to help anyone.” (Interview 11)
	Other preparation related to programming	“When I’m…onboarding someone [newly hired CHW] I have them to understand their clear role of navigation, advocate for patient. ‘You’re the eyes and ears for that position. You’re their eyes and ears to help them introduce them into the medical health system.’” (Interview 2)
		“Having all these documents that you're going to be giving them [clients] or reviewing them as well to get you know each document was also very helpful.” (Interview 5)
		“I took the responsibility… to learn a little bit more English, I try my best I can and because is the base you want to serve, not just my community… as soon as I can speak English a little bit to get my message, I think we doing the best.” (Interview 9)
Characteristics of community health workers	Qualities needed in the field	“I'm very patient, I am very flexible. I have a level of passivity that doesn't garner conflict or pushback, so I think that helps with work relationships and workspaces.” (Interview 6)
		"Respect their [clients’] beliefs and their culture it’s very important.” (Interview 8)
		“You’ve got to be open to work with all kinds--homeless, intoxicated people, people in a bad mood. You need to be empathetic... It is still your duty to help them.” (Interview 8)
		“What has made me effective as a CHW is I’m a people person. I love talking to people. I love interacting with new people. It’s very easy for me to connect with people that I have never met before, so that’s been very helpful. I think people can sense when you’re genuine and when you actually want to help them, so it makes it a lot easier to connect.” (Interview 11)
		“I would say my compassion. This [community health work] field is my compassion to help others. As a young lady myself, my mother and my father were older. I felt like I didn’t have much of a mentor that was my age. I always had someone older than me. When I see people that were the situation like myself, if I’m working with younger people or young women and particularly young women with babies or wanting to have babies, my heart goes out to them. And I just have a compassion for helping women and children.” (Interview 13)
	Skills needed in the field	“You may be managing 20 to 30 cases. And right now I'm only managing eight, so being able to take care of all the needs of each patient and also give them the attention that they need.” (Interview 4)
		“At the beginning, I wanted to do it all. I thought, ‘I could do this, I could do that.’ And then, it was causing burnout. I was working more than I should have... And saying no as well, was something that just made me more efficient [and] successful.” (Interview 5)
		“Clear communication, time management as much as I can. Some detective skills, the ability to connect and trace and search for things is an important skill.” (Interview 6)
		“I think part of being a community worker is to be resourceful. You have to do your job in whatever setting environment you have. So being resourceful is definitely key.” (Interview 7)
		“It’s different communicating with them [clients] than it is communicating with you. You have to be able to speak to anyone on any level - street level, education level...You’ve got to be able to deal with any type of person.” (Interview 12)
		“My biggest thing is communication. With our clients, you have to be able to communicate with them for them to understand. I don’t want to sound negative. You have to come down to their level and the way that they speak to you, you’re going to have to speak that way back. You speak to them the way that they speak to you in order for them to understand what you’re talking about. Have you ever been in a situation where you hear somebody talking and you really don’t understand what they’re talking about? Well, you have to fit yourself in those situations where you could talk to them the way that they talk to you, and they can understand it better.” (Interview 12)

#### Theme 1: Background of CHWs

Participants highlighted innate attributes to consider when hiring CHWs. One characteristic considered important was being a member of the community served. Participants shared this quality was valuable for navigating local resources and building trust with clients. Participants emphasized the importance of having shared backgrounds (e.g., demographics, experiences) and language with clients to build trusting relationships. One participant shared that having the same adversities as clients prepared them to join the workforce, *“I was the mother waiting in a room to get the service, and nobody gave the service because [there was] nobody who speaks Spanish... I'm not the first, but I'm not the last one. That gave me that responsibility.”* Along with shared backgrounds as clients, participants reported certain past experiences that transferred to their roles, including education in psychology or sociology as well as previous roles in health or helping others.

#### Theme 2: Champions to support the work of CHWs

Participants listed key players who have supported their role as CHWs. An essential entity identified by participants as a champion was their own internal organization. They expressed appreciation that their organization’s culture encourages collaborations and nurtures professional growth by providing education and materials. One participant shared about their organization, *“I think one of the reasons that I really like to do what I do is because I feel supported and because, like [organization’s] philosophy, if we’re okay emotionally, we can better perform our job.”*

Internal to their organizations, participants reported their CHW teams were champions for their work. Their team members were especially helpful by providing guidance and emotional support, particularly when starting in their roles. One participant described their team member’s lasting impact when beginning their role, *“Something came up...I’m like, ‘I can’t do this. I’ve got to find something else to do.’ My [team member] was like, ‘Okay, see you tomorrow’… When people new come to me, I’m like, ‘Okay, let’s take a deep breath. Go home and do some self-care. I’ll see you tomorrow. Tomorrow, let’s talk about what happened yesterday.’”* In addition to serving as champions at the start, participants emphasized that team members helped enhance their performance by sharing expertise and resources as well as creating space to debrief on difficult cases. Lastly, participants stated the leaders within their teams are champions for their work, providing support and resources for CHWs to be effective in their role. One participant shared the importance of having a leader who respects them, *“Having that type of support with your supervisor does make that type of work a lot easier...it makes [me] happy to be working in that field.”*

Outside of their organizations, some participants recognized external organizations as champions for their work. Participants shared that they relied on other CHW-focused organizations for topic expertise and client referrals. Participants who have been integrated in clinical settings emphasized the importance of having a clinician who trusts the CHW and serves as a champion. One participant shared their experience, *“I have this one champion...she is a doctor still. She is the one that was, ‘go ahead and talk [to] them,’ ‘go ahead and do this,’ ‘go ahead and tell them.’”*

#### Theme 3: Materials to perform work of CHW

Participants highlighted the various materials needed in their line of work. A key material was readily available resources with information and referrals for clients, such as community referral programs like “Purple Binder” and “NowPow”. One participant described how they use such resources with clients, *“I have physical resources. I can get copies and highlight numbers they’ve got to call, addresses, appointments.”* Participants also shared that CHWs need materials for client care, including medications, medical supplies, and cleaning products to support chronic condition management at home. During the COVID-19 pandemic, they also provided personal protective equipment and at-home tests. Further, participants discussed that communication tools were important for working with clients and their teams, including phones, social media, and translated materials for non-English speaking clients. For their data collection responsibilities, CHWs typically used laptops or tablets to record their work and as appropriate database software. Participants also suggested CHWs should have paper versions of documents in times without Wi-Fi access. To support client education, participants recommend CHWs should have presentations as well as visual tools like anatomical models and practical materials (e.g., diabetes and asthma devices) to demonstrate aspects of disease management. As for continued education, participants largely relied on online tools like Coursera and YouTube.

#### Theme 4: Preparation for CHW role

Participants reported training and continuing education were essential to be prepared for the CHW field. Specifically, participants highlighted the various lessons from CHW core skills training, which focuses on relevant competencies and skills that were applicable when working closely with diverse clients. One participant shared they felt prepared for the CHW role after completing such training, *“I had to understand culture humility...how to communicate with physicians...how to document... Once the expectation had been set and the knowledge has been layered on that, then it gets you ready to do the work in hand.”* In addition, participants reported trainings on various health topics prepared them to support condition-specific programs. Some participants were cross-trained on multiple health topics, including chronic health conditions (e.g., asthma, diabetes, breast cancer), mental health, and COVID-19. These trainings ensured that CHWs attained knowledge on health topics to share with clients, as described by one participant, “*We can all jump in different interventions and educate a person. If I’m seeing somebody for breast health and I know that person has asthma, I can interject some of my asthma knowledge.*” Along with CHW core skills and health topic trainings, participants emphasized the importance of having practical experience through role-playing, shadowing experienced CHWs, and learning on the job. One participant shared, “*If you don’t have experience from doing home visits with the educators…having face-to-face contact with people… you're never going to have that connection with people [clients].*” Also, participants emphasized the importance of continuing education to provide quality services to clients. Participants described they have requested subject-specific training on health topics and skills from their internal organization as well as conducted their own research or completed online trainings to ensure up-to-date knowledge.

Along with trainings, participants shared it is vital for individuals to complete other preparation for their role, including developing familiarity with the people and needs of communities they are serving. In addition, some participants emphasized the importance of knowing the community for their safety, as one participant shared, *“You can’t help anyone if you’re not around to help anyone.”* Finally, some participants highlighted additional preparations related to programming, such as reviewing materials and understanding responsibilities.

#### Theme 5: Characteristics of CHW

Participants listed qualities and skills that individuals should have to work in the CHW field, some of which are gained or refined through external experiences and preparation for their CHW role. They commented that key qualities of a CHW include being respectful, flexible, and compassionate. Participants emphasized the importance of being comfortable working with others when taking on the role of CHW, as one participant stated, *“People can sense when you’re genuine and when you actually want to help them.”* In addition, participants commented that it is important to be empathetic, especially when working with various clients with different backgrounds. One participant stated, *“You’ve got to be open to work with all kinds--homeless, intoxicated people, people in a bad mood. You need to be empathetic... It is still your duty to help them.”*

Regarding skills necessary to be an effective CHW, participants highlighted resourcefulness, interpersonal skills, problem solving, and time management. They emphasized the importance of having strong communication skills to work with various parties – clients and individuals within both internal and external organizations. One individual stressed the importance of tailoring communication style for clients with various backgrounds, *“You have to be able to speak to anyone on any level - street level, education level...You’ve got to be able to deal with any type of person.”* In addition, participants commented that it is important for CHWs to be mindful of their limitations and create healthy boundaries in their role, *“At the beginning, I wanted to do it all. I thought, ‘I could do this, I could do that.’ And then, it was causing burnout…saying no as well was something that just made me more efficient [and] successful.”*

## Discussion

As CHWs serve increasingly important roles in connecting health care, social care, and community, this study provides insights into the current work of CHWs from the perspective of experienced workforce members who are set within Chicago’s unique environment. Further, this study is the first to utilize such perspectives to develop a logic model that delineates key early strategies for CHWs to succeed in their role and how organizations can support these early stages.

Participants highlighted that current CHW responsibilities focus on two areas: client care and workforce sustainability. For client care, they described various roles in which CHWs support community and individual needs, in alignment with current literature. Examples include conducting health assessments, providing local health and social resources, and educating about health [3, 9, 28]. To effectively deliver these services, CHWs must build trusting relationships with clients, coworkers, and community members. This study’s findings corroborate existing literature that describes the critical roles of CHWs in healthcare and community settings, including establishing unique connections with patients to inform clinical teams on tailoring care for unique needs and gaining trust of community members to deliver tailored interventions within intimate settings (e.g., home visits) [5, 20, 29–31]. These capabilities and strengths of CHWs should be considered when designing CHW interventions within clinical, community, and broader settings to maximize the impact of CHWs on patient, community, and workforce outcomes. Programming should also take into account the challenges raised by participants related to building trust with clients and the community, including the mistrust of health providers and healthcare systems as well as limited resources [32, 33]. These barriers can contribute to distress or demoralization among CHWs [32, 33]. As such, along with programmatic efforts to alleviate these barriers, a range of approaches should be adopted to support the wellbeing of CHWs. Participants’ recommendations and prior research suggest CHW support systems, self-management trainings, and coaching on strengthening engagement, while also establishing and maintaining healthy boundaries to avoid burnout in the role, are critical to success and sustainability [32, 34].

While CHW responsibilities related to client care are well-established in the literature, their roles in promoting workforce sustainability have been underexplored. In this study, CHWs described their roles in programming, including developing materials and training for CHWs. These activities play a significant role in sustainability and are not traditionally recognized as core responsibilities for CHWs [20]. With unique insights from their on-the-job experiences, senior CHWs have contributed to improving training standards by updating training curricula as well as leading training sessions [35]. Further, this study also highlighted CHWs’ involvement in program evaluation and quality assurance, which has been described in literature [20, 36, 37]. CHWs are often responsible for collecting data on client interactions and outcomes, which is utilized to evaluate and improve programs as well as to secure funding [38]. Beyond data collection, involving CHWs in all stages of evaluation and research, from identifying research questions to disseminating findings, has been recommended [38]. In addition to material development, training, and program evaluation, a few CHWs in our study cited involvement in program design. Such opportunities can be foundational to developing successful programs for clients and further building the workforce [39]. Existing programs can consider expanding CHWs’ involvement in program development and evaluation to incorporate their valuable perspectives into such efforts.

Based on the multiple CHW responsibilities described, this study’s findings informed a new logic model that outlines key resources and activities essential for the early success of CHWs (Fig. 1). While some aspects of this logic model align with existing research, this model moves beyond to examine resources and activities across various levels. Existing frameworks in the literature focus primarily on CHW workforce readiness, including trainings, evaluation, and broader program support [35, 40, 41]. For instance, a pre-existing logic model for CHWs describes programmatic and systematic factors that enhance CHW performance in low and middle-income countries; however it does not incorporate factors at the individual level [41]. This study’s logic model incorporates factors across individual, interpersonal, program, and systems levels that are critical for the effectiveness of CHWs in the early stages of their roles. Organizations that are developing CHW programs can reference this logic model to understand the necessary infrastructure and resources required to set a CHW for success in their role. Additionally, this model can be utilized for established programs to identify gaps and implement strategies to effectively support CHWs in their programs.

Informed by participants, the inputs of this model are the background of CHWs, champions to support CHWs, and materials to perform the work—key factors at the individual level. Participants in our study noted it is important to select CHWs from the communities being served who share experiences with community members. This finding corresponds with systematic literature reviews showing that CHWs residing in local communities understand community culture and language(s) in unique ways, which enable them to establish and build trust and respect among clients [28, 30]. While participants reported that educational background in certain subjects prepared them for the CHW workforce, there is no existing consensus on criteria for level of education. Qualifications for the CHW role in current literature have varied widely, from high school to secondary education, in addition to relevant training courses [3, 30]. In terms of prior work experiences, this study mirrors prior research which has documented the importance of experiences in the health or social welfare sectors, such as a caregiver or community organizer [28]. The considerations outlined can be valuable for organizations initiating or expanding CHW programs, particularly when hiring qualified candidates for CHW positions.

In addition to the background of CHWs, additional inputs of the model are ensuring there are champions to support CHWs as they begin and carry out their work as well as materials to perform the work. Opportunities to support CHWs include providing essential materials for fulfilling their responsibilities as well as creating systems to ensure mentorship in their work [42, 43]. Beyond the opportunities mentioned in this study, literature has described that job aids (e.g., checklists, pictorial instructions) and transportation options are useful to support CHW activities [44, 45]. Additionally, prior research has identified that access to electronic health records is helpful in identifying clients, scheduling appointments, and facilitating communication between clients and care teams [42]. Lastly, collaborations between CHWs and their CHW teams can be a source of valuable support, a finding consistent with previous studies that highlight the benefits of CHW supervision and peer support [42, 43]. While adopting strategies for supportive supervision for CHW programs, such as supervisors coaching and mentoring CHWs, is not strongly recommended, [46] organizations that integrate CHWs should ensure to hire and train supervisors to offer comprehensive support to CHWs. This recommendation stems from participants’ emphasis on the impact of their CHW team leader’s feedback and advocacy. These considerations can help organizations implement an infrastructure of support for CHWs to succeed in their work with the necessary equipment and robust support system.

Building upon the inputs in the logic model, participants highlighted key activities for CHW success, including completing trainings as well as deepening insights and connections within communities. Suggested training topics include core competencies (e.g., cultural humility, advocacy) and health topics (e.g., mental health), which corresponds with topics described in the literature [35, 47]. Along with trainings, CHWs should gain practical experience and community familiarity to succeed in their roles through activities like role-playing as well as conducting research to identify health or social issues within communities and develop plans to address them [35, 48]. While the training and experience can support preparedness for the role, CHWs’ knowledge and skill proficiencies can also be evaluated using observation, examination, and self-assessment [35, 40, 49]. Such strategies can equip CHWs with the qualities and skills identified as crucial for success, such as emotional intelligence, cultural competence, interpersonal skills, and problem-solving abilities–the outputs of our logic model. These qualities and skills are frequently discussed in existing literature as key competencies for CHWs, along with characteristics such as an open-minded personality and respect for diversity [50]. Other attributes in research that were not emphasized by this study’s participants include intrinsic motivation and soft skills like leadership [28, 30, 50]. It is critical for CHW organizations to provide such learning opportunities for newly hired CHWs to acquire the necessary skills and knowledge to be effective in their role as well as to offer continuous professional development as the role and field evolve.

Strengths of this study include the rich, comprehensive data from one-on-one interviews with participants and the prioritization of the perspectives of CHWs, who possess unique insights into their role. The generalizability of the findings may be limited as all participants were from the Chicago area, and experiences may differ in non-urban or rural areas, US states, and countries with different policies about community health work. Most participants worked within healthcare systems, providing insights relevant to a sizeable proportion of CHWs; however, these experiences may not be shared by CHWs without such affiliations [3, 51]. Also, interviews may have been affected by social desirability bias or recall bias. The researchers attempted to minimize bias by ensuring the interviewer was not affiliated with the participants’ organizations, asking participants questions about various experiences, and de-identifying transcripts prior to analysis. Future steps include gathering more experiences from CHWs with different backgrounds, roles, and/or programs to validate the framework.

It is also important to recognize this study’s findings focus on preparing a CHW and early strategies for success. It does not contemplate the longer-term support needed as the work of CHWs evolves over time. Experiences show that effective supervision, consistent upskilling opportunities, peer support and learning, and intentional career ladders are all important to the longer-term success of CHWs [52].

## Conclusion

This study sheds light on the role of CHWs in improving the health of vulnerable communities and sustaining the workforce in Chicago. Current responsibilities that contribute to such efforts include providing services to clients and collaborators along with building relationships, collecting data, and overcoming challenges related to their role. In addition, this study provides a framework for community-based organizations and policymakers to apply to future CHW programming to ensure that qualified CHWs are supported and equipped to be successful in their role with the necessary trainings, materials, and support systems. As the CHW workforce is expanding, it is essential to recognize and leverage CHWs’ current abilities and enhance their effectiveness to achieve health equity among vulnerable populations.

## Data Availability

No datasets were generated or analysed during the current study.

## Abbreviation

CHW: Community health worker
